# Acupuncture in trauma-related disorders – the novel treatment approach with acupuncture-based exposition (ABE) method

**DOI:** 10.1192/j.eurpsy.2025.510

**Published:** 2025-08-26

**Authors:** A. Sobieraj Bunse, J. Schottdorf, R. Musil

**Affiliations:** 1 Ludwig Maximilian Universität, Munich; 2 Privatklinik Oberberg, Bad Toelz; 3 Private Practise, Friedberg, Germany

## Abstract

**Introduction:**

The evaluation of trauma-related disorders is becoming increasingly significant in psychiatric assessments. According to the DSM-5, these disorders are categorized as Post-Traumatic Stress Disorder (PTSD), Acute Stress Disorder, Adjustment Disorders, Prolonged Grief Disorder, Reactive Attachment Disorder, and other specified trauma- and stressor-related disorders. International guidelines on post-traumatic symptoms report positive outcomes with trauma-focused cognitive-behavioral therapy and EMDR, as well as non-trauma-focused therapies, such as relaxation techniques. Acupuncture and acupressure techniques can be used not only for treating symptoms but also for addressing associated sleep disorders, headaches and affective disorders. These methods are clinically applied to patients with traumatic events, primarily as ear acupuncture using the NADA protocol, along with ‘Battlefield Acupuncture,’ which has been established among American soldiers. In our clinical work, we use a unique protocol called Acupuncture-Based Exposition (ABE) (Schottdorf & Musil, 2017).

**Objectives:**

The assessment of the effectiveness of the newly developed method of Acupuncture-Based Exposure (ABE).

**Methods:**

The ABE (Acupuncture-Based Exposition) method was developed by Dr. Schottdorf and investigated by Dr. Musil and has already been clinically applied in their practice and clinic to a variety of patients. It has also been invetigated in an initial pilot study focusing on Type I trauma-related disorders, post-embitterment disorder as well as postpartum depression, and pain disorders. During ABE sessions, after a basic acupuncture treatment, patients are guided to visualize images of their traumatic experiences. The resulting physical sensations and emotions are addressed through corresponding acupuncture points until their intensity decreases. Finally, the imagined images are faded out using points on the head. Patients subsequently report a significant reduction in the burden of intrusive memories.

**Results:**

In an initial case series involving 24 patients with trauma-related symptoms, an average reduction in trauma-specific symptoms was observed after just 3.95 sessions, as measured by the Impact of Events Scale-Revised (IES-R), from 55.6 ± 23.0 to 16.2 ± 21.1 (Wilcoxon test: p < 0.002). Additionally, a decrease in depressive symptoms was noted, measured by the Beck Depression Inventory, from 38.3 ± 8.0 to 25.6 ± 8.0 (Wilcoxon test: p < 0.001) (Schottdorf, 2018). So far, there have been no investigations into the mechanisms of action of ABE beyond clinical experience and initial data from clinical pilot projects.

**Conclusions:**

Particularly, integrating acupuncture as an adjunct to conventional treatments for mental disorders appears to offer promising results. Further clinical research in the field of acupuncture and the ABE method is necessary.

**Disclosure of Interest:**

None Declared

